# Motives for Caffeine Consumption Questionnaire (MCCQ): validity and reliability of Turkish version

**DOI:** 10.1186/s41155-023-00285-9

**Published:** 2024-01-03

**Authors:** Tuğba Küçükkasap, Burcu Uslu

**Affiliations:** 1 Department of Nutrition and Dietetics, Faculty of Gulhane Health Sciences, University of Health Sciences, Gülhane Complex, Emrah Neighborhood, Etlik/Keçiören/Ankara, 06018 Türkiye; 2 Department of Nutrition and Dietetics, Faculty of Health Sciences, Yuksek Ihtisas University, Oğuzlar Neighborhood, 1375 Street, No.: 6, Balgat/Ankara, Turkey

**Keywords:** Caffeine use disorder, Motives for caffeine consumption questionnaire, Caffeine, Reliability and validity

## Abstract

**Objectives:**

The aims of this study are to make the Turkish validity and reliability of the scale that explain why caffeine intake. Additionally, it is crucial to highlight that the adaptation of the scale involved a confirmatory factor analysis (CFA) to validate its structure and the need for this adaptation in the Turkish context.

**Materials and methods:**

The study was carried out with a total of 200 university students, comprised of 93.5% female and 6.5% male participants, with a mean age of 21.74 ± 6.15 years who were actively studying in the faculties of the universities who accepted to participate voluntarily in the study were included. The Turkish version of the Motives for Caffeine Consumption Questionnaire (MCCQ) scale and the MCCQ was used as the data collection tools. To evaluate the statistical analysis findings, the margin of error was taken as 5%. The entire application was carried out with the R-project software. CFA was used to test the adaptation of the MCCQ scale from English to Turkish.

**Results:**

The alpha internal consistency coefficient for the whole scale was calculated as 0.959. Just like the original scale, the Turkish version shows a six-factor structure: habit, alertness, mood, social, taste, and symptom management. In MCCQ-TR, it showed a low level of positive correlation with Yale Food Addiction Scale (YFAS). For the final scale with 23 items and 6 sub-dimensions, it was determined that the fit indices were at an acceptable level, and the internal consistency was highly reliable for all sub-dimensions and the total scale.

**Conclusions:**

It has been determined that the Turkish version of the MCCQ is a valid and reliable measurement tool to operationalize the motives of caffeine consumption and to reveal possible differences in the motives regarding gender, age, and the type of caffeinated products consumed. The use of CFA in adapting the scale ensures the robustness of the structural validity in the Turkish context, making this adaptation a valuable contribution to the field.

## Introduction

Caffeine is the most commonly consumed psychoactive substance in the world. Caffeine is not only found in coffee but also present in other products that include tea, energy drinks, caffeine-containing medications, and also in soft drinks. On a daily basis, about 80% of the world’s population consumes some form of caffeine (Rajeswaran et al. 2020). Studies indicate that caffeine, which has a rejuvenating effect, can act as a diuretic, affect the body fluid balance, and cause impaired coordination, irritability, insomnia, and palpitations in case of excessive consumption. It has been determined that caffeine consumption reduces the absorption of vitamins and minerals with its diuretic effect and prevents the use of calcium in the body. Consumption of caffeine can cause hypertension. It is very important to evaluate caffeine consumption due to factors such as excessive caffeine consumption that prevent the normal functioning of the central nervous system (Küçükkömürler & Kurt, 2018). Caffeinated beverages have gained increasing popularity, especially among young generations, as lifestyles have become more westernized. Food and beverage consumption trends indicate coffee is a preferred food rather than a functional food containing caffeine. However, most consumers are not aware of the amount of caffeine in their caffeinated drinks or its effects on them (Choi, 2020). In the college student population, there is concern of overuse (> 400-mg caffeine per day FDA recommendation; > 4 standard cups of coffee), particularly because of the increased popularity of energy drinks (75 to 174 mg caffeine per serving) (Driver et al. 2020). Caffeine use disorder is not currently a recognized psychiatric condition, but caffeine withdrawal is included in the DSM-5. The prevalence of caffeine withdrawal is estimated to be between 10 and 55% in the general population of caffeine user. The emergence of symptoms occurs after a period of prolonged use resulting in significant distress or impairment. These symptoms include headache, fatigue or drowsiness, and difficulty concentrating, irritable or depressed mood, and flu-like symptom. Withdrawal symptoms usually commence within 12–24 h of abruptly stopping caffeine consumption and last between 2 and 9 days. It may be, however, that the DSM-5 withdrawal diagnostic criteria are too narrow (Rodda et al. 2020). Gaining a greater understanding of the motivations for caffeine consumption is important for experts in the health sciences and nutrition. These motivations may be associated with the intensity and persistence of caffeine consumption and may help us understand caffeine dependence or abuse. Therefore, the validity of the Caffeine Consumption Motives Questionnaire and its relationship with other associated variables, especially the Yale Addictive Food Questionnaire, should be better understood. In this context, this study will also address the theoretical framework of the Caffeine Consumption Motives Questionnaire. Furthermore, the results of the Caffeine Consumption Motives Questionnaire will be compared with the results of other variables, in particular the Yale Dependence Food Questionnaire, in order to examine potential relationships between the results of the Caffeine Consumption Motives Questionnaire and the results of other variables. Caffeine dependence occurs as a combination of psychological and physiological factors. Caffeine consumption has been associated with positive effects such as alertness, increased concentration, and energizing. However, excessive caffeine consumption also has negative effects, further complicating the phenomenon of addiction. Therefore, the assessment and understanding of caffeine dependence are of great importance. In this context, measurement tools such as the “Caffeine Consumption Motives Questionnaire” have been used to examine motivation for caffeine consumption. However, further studies on the validity and reliability of such scales are needed. The aim of this study is to adapt the “Caffeine Consumption Motives Questionnaire,” a scale that can be used to assess caffeine consumption motivation, into Turkish and to examine the psychometric properties of the scale.

The aim of this study was to verify the use of caffeine in adults in Turkey for that reason and to test the validity of MCCQ motivation and reliability. It was aimed to reveal the motivation of caffeine consumption in order to draw attention to this deficiency in the literature and to create reference data.

## Methods

The ethics committee permission required for the study was obtained by the Ethics Committee of Istanbul Okan University on 08.01.2020 with the permission of the ethics committee number 117. Demographic information of individuals such as age, height, and weight was recorded.

### Design and participants

This methodological research was conducted in the Faculty of Health Sciences of a state university in the capital city of Turkey. Research population consisted of students aged 18 years or older who were actively studying in the faculties of the universities specified between 30 December 2019 and 30 June 2020. The sample of the study was calculated by performing power analysis with the Raosoft Sample Size Calculator, and it was deemed appropriate to include at least 188 volunteer students in the study. The students who met the criteria for inclusion in the study were selected by using the nonrandom sampling method. Criteria for inclusion in the study were being 18 years old or older, studying in Faculty of Health Sciences, and agreed to volunteer in the study. The participants included in the sample consisted of university students actively studying in various health sciences faculties. These students are studying medicine, nursing, physiotherapy, dentistry, and other health-related departments.

### Instruments

#### Descriptive characteristics and sociodemographic form

This form consisted of questions that determined the demographic characteristics of the students (age, sex, and so forth), their nutritional habits, and their anthropometric measurements (weight, height). In sociodemographic information, age, gender, and living situation with the family were questioned. Health information included the question of whether they had any chronic diseases diagnosed by a physician. Individuals’ height (m) and body weight (kg) were questioned on a self-reported basis.

#### Yale food addiction scale

Before starting the Yale scale consisting of 25 questions, the students were asked to mark the foods that they had difficulty controlling from time to time. These foods are sweet foods such as ice cream and chocolate, starchy foods such as white bread, savory snacks such as chips and crackers, and fatty foods such as hamburgers and French fries. It is divided into five categories as sugary drinks such as soda. The first 16 questions in the scale are 5-point Likert type and are scored between 0 and 4 points. The answers given to the questions are in the form of yes/no and are scored between 0 and 1 point. In the 25th question that follows, it is questioned how many times have been tried to reduce or stop eating certain foods in the last year. Questions 17 and 18 are not scored as they are premises for other questions. In order to determine the diagnostic criteria, the questions were divided into seven groups. The score for each diagnostic criterion is calculated separately. The total score ranges from 0 to 7. In determining food addiction, questions 15 or 16 are clinically important, a score of 1 should be taken, and a diagnosis of food addiction is made when the number of symptoms > 3 (Gearhardt et al. 2009). The scale was adapted to Turkish for clinical and nonclinical groups by Bayraktar et al. and the Cronbach alpha value was found to be 0.93. The Yale Food Addiction Scale consists of subscales measuring various dimensions of food addiction.

- Habit: This subscale assesses whether food habits are repeated and regular. Cronbach’s alpha reliability coefficient is 0.858.
- Alertness: This subscale measures whether food addiction increases alertness. Cronbach’s alpha reliability coefficient is 0.947.
- Mood: This subscale assesses whether food addiction affects emotional state. Cronbach’s alpha reliability coefficient is 0.921.
- Social (social relationships): This subscale measures how food addiction affects social interactions. Cronbach’s alpha reliability coefficient is 0.880.
- Taste: This subscale assesses the role that taste expectations play in food intake. Cronbach’s alpha reliability coefficient is 0.912.
- Symptom management: This subscale measures the purpose of consuming certain foods in managing withdrawal symptoms. Cronbach’s alpha reliability coefficient is 0.575 (Bayraktar et al. 2012).

### Process of cultural adaptation

The back-translation method was also applied in the translation process of the questionnaire. First, the questionnaire was translated into Turkish by three different people who are fluent in English and have a good command of both languages. Then, after the translation was completed, the questionnaire was back-translated by five different experts in order to get their opinions and suggestions on the comprehensibility and appropriateness of the Turkish translation. In the retranslation phase, the original version of the questionnaire and its translation were sent to the experts together, and the scale was re-evaluated, and necessary corrections were made on the items based on the opinions and suggestions received for each item through the “Expert Evaluation Form” sent to the experts who would make the evaluation. Finally, the questionnaire was back-translated by a linguist whose mother tongue is Turkish and who has no knowledge on the subject, taking into account the information in different sources. After all these steps were completed, the questionnaire was finalized and written for use in the study. These methods aimed to ensure that the questionnaire was translated accurately and meaningfully, and that it was appropriate.

### Statistical analysis

The descriptive statistical findings of the Motives for Caffeine Consumption Questionnaire developed in the first stage and the Yale Food Addiction Scale used in the research are presented. The mean (Mean), standard deviation (SD), minimum (Min), and maximum (Max) values of descriptive statistics are shown together. In the next step, Cronbach’ alpha reliability analysis was applied to examine the internal consistency of the developed scale. The descriptive statistics of the items of the scale developed with the findings of the reliability analysis are given. Then, confirmatory factor analysis (CFA) was applied to test the validity of the developed scale. Diagonal weighted least squares (DWLS) technique was preferred since the data were Likert type in the estimation phase of CFA. In the last stage, the relationship between the Yale Food Addiction Scale and the scale was examined in order to test the convergent validity of the developed scale. At this stage, the conformity of the measurement scores to the normal distribution was evaluated with the Shapiro–Wilk test, and the relationship between them was determined with the Pearson correlation test.

To evaluate the statistical analysis findings, the margin of error was taken as 5%. The entire application was carried out with the R-project software (R Core Team, 2022). The lavaan package in the R program was used in the analysis process (Rosseel, 2012).

## Results

### Descriptive statistics

Table 1 shows the descriptive statistics values of the sub-dimensions and total scores of the Yale food addiction and Motives for Caffeine Consumption Questionnaire of the 200 participants who participated in the study. When the descriptive statistics values were examined, the Yale Food Addiction Scale total score average was found to be 0.493 ± 0.294. In addition, individuals’ habit sub-dimension mean score is 2.990 ± 1.370, alertness sub-dimension mean score is 2.592 ± 1.068, mood sub-dimension mean score is 2.663 ± 1.292, social sub-dimension mean score is 2.535 ± 0.965, taste sub-dimension mean score is 3.475 ± 1.233, symptom management sub-dimension mean score was 1.900 ± 1.001, and total Motives for Caffeine Consumption Questionnaire mean score was 2.692 ± 0.964.

**Table 1:** Descriptive statistics of individuals’ sub-dimensions and total scores of Yale food addiction and motives for caffeine consumption questionnaire

Variable	Mean	SD	Min	Max
**TSEE**	0.400	0.808	0.000	3.000
**ORR**	0.945	0.603	0.000	2.000
**STMTP**	0.295	0.728	0.000	3.000
**GDS**	0.265	0.773	0.000	4.000
**CUBR**	0.650	0.478	0.000	1.000
**TD**	1.035	0.726	0.000	2.000
**CWS**	0.180	0.582	0.000	3.000
**UCCI**	0.175	0.464	0.000	2.000
**Yale total**	0.493	0.294	0.000	1.875
**Habit**	2.990	1.370	1.000	5.000
**Alertness**	2.592	1.068	1.000	5.000
**Mood**	2.663	1.292	1.000	5.000
**Social**	2.535	0.965	1.000	5.000
**Flavor**	3.475	1.233	1.000	5.000
**Symptom management**	1.900	1.001	1.000	5.000
**Caffeine total**	2.692	0.964	1.000	4.917

*Mean* mean, *SD* standard deviation, *Min* minimum, *Max* maximum, *TSEE* taking the substance in excess for longer than expected, *ORR* ongoing request or repeated failures to quit, *STMTP* spending too much time in procurement, use, and overcoming, *GDS* giving up or decreasing social, occupational, and leisure activities, *CUBR* continuing to use even though it is known to have bad results, *TD* tolerance development (in effect decrease, increase in amount), *CWS* characteristic withdrawal symptoms, substance use to reduce withdrawal symptoms, *UCCİ* Use to cause clinically significant impairment

### Reliability analysis

Table 2 shows the descriptive statistics and Cronbach’s alpha reliability analysis results obtained from the subscales of the Motives for Caffeine Consumption Questionnaire of individuals. When the reliability analysis findings are examined, it is seen that there is no significant increase in the reliability coefficient when the items are removed for the subscales of the Motives for Caffeine Consumption Questionnaire of individuals. However, the corrected correlation values of the subscale items of the individuals’ Motives for Caffeine Consumption Questionnaire were found to be positive. In the light of these findings, the Cronbach’s alpha coefficients for the general and sub-dimensions of the individuals’ Motives for Caffeine Consumption Questionnaire were determined to be 0.959, 0.858, 0.947, 0.921, 0.880, 0.912, and 0.575, respectively.

**Table 2:** Reliability analysis of individuals’ motives for caffeine consumption questionnaire

Dimension	Substance	Avg	SD	CC	AID	Alfa
**Habit**	K1	3.035	1.430	0.812	0.718	0.858
	K8	2.945	1.498	0.812	0.788	
**Alertness**	K2	2.815	1.326	0.835	0.939	0.947
	K4	2.735	1.266	0.832	0.939	
	K9	2.625	1.365	0.838	0.939	
	K11	2.620	1.201	0.639	0.950	
	K13	2.500	1.315	0.828	0.940	
	K16	2.300	1.199	0.782	0.942	
	K18	2.391	1.251	0.796	0.941	
	K20	2.675	1.288	0.904	0.936	
	K22	2.660	1.298	0.878	0.937	
**Mood**	K6	2.715	1.328	0.890	0.836	0.921
	K12	2.610	1.355	0.890	0.872	
**Social**	K7	1.915	1.133	0.512	0.891	0.880
	K10	2.130	1.204	0.802	0.851	
	K14	2.225	1.201	0.829	0.846	
	K15	3.205	1.297	0.757	0.860	
	K19	2.421	1.258	0.766	0.853	
	K21	3.320	1.243	0.797	0.852	
**Taste**	K3	3.545	1.283	0.878	0.835	0.912
	K17	3.405	1.288	0.878	0.842	
**Symptom management**	K5	2.235	1.345	0.546	0.561	0.575
	K23	1.553	1.007	0.546	0.315	

*AVG* average, *SD* standard deviation, *CC* corrected correlation, *AID* alpha when item was deleted

### Confirmatory factor analysis (CFA)

GFI, AGFI, CFI, TLI, RMSEA, and SRMR values, which were not affected by the study scope estimates and parameter estimates, were examined. Various benefits were used to decide on good fit values and acceptable values. The acceptable fit value for CFI indices is 0.90, and the perfect fit value is 0.95. For RMSEA, the acceptable fit value is 0.10, and the perfect fit value is 0.5. Acceptable fit value for SRMR is 0.05–0.10, and perfect fit value is 0.00–0.05. Acceptable fit value and perfect fit value > 0.50 are accepted for AGFI indices. The fit index values of the correct factor analysis measures of the individuals’ caffeine use motivation data are evaluated in Table 3. The fit index values show that the chi-square statistic/*SD* = 0.752 value is below 2, the GFI, AGFI, CFI, and TLI values are above 0.950, RMSEA officers are below 0.05, and SRMR officers are below 0.08. When the fit index values are evaluated together, the validity results for the caffeine consumption motivation system of the individuals indicate perfect fit.

**Table 3:** Fit indices of CFA findings of individuals’ caffeine consumption motivation scale

Chi-square (de)	GFI	AGFI	CFI	TLI	RMSEA	SRMR
161.680 (215)	0.991	0.989	1.000	1.000	0.000	0.060

*de* degrees of exemption

CFA statistics of individuals’ Motives for Caffeine Consumption Questionnaire are shown in Table 4. When the CFA statistical findings are examined, it is seen that all subitems of the individuals’ Motives for Caffeine Consumption Questionnaire are statistically significant (*p* < 0.05).

**Table 4:** CFA statistics of individuals’ motives for caffeine consumption questionnaire

Dimension	Substance	Beta	SE	*z*-statistics	*p*
**Habit**	K1	1			
	K8	1.118	0.040	27.776	< 0.001
**Alertness**	K2	1			
	K4	0.861	0.032	26.568	< 0.001
	K9	1.029	0.036	28.266	< 0.001
	K11	0.572	0.028	20.543	< 0.001
	K13	0.982	0.035	27.809	< 0.001
	K16	0.744	0.030	25.161	< 0.001
	K18	0.823	0.032	25.618	< 0.001
	K20	0.965	0.035	27.843	< 0.001
	K22	0.950	0.035	27.334	< 0.001
**Mood**	K6	1			
	K12	1.064	0.039	27.074	< 0.001
**Social**	K7	1			
	K10	2.650	0.277	9.551	< 0.001
	K14	2.536	0.268	9.452	< 0.001
	K15	3.402	0.346	9.830	< 0.001
	K19	2.832	0.294	9.634	< 0.001
	K21	3.327	0.337	9.864	< 0.001
**Taste**	K3	1			
	K17	0.950	0.036	26.673	< 0.001
**Symptom management**	K5	1			
	K23	0.519	0.032	16.110	< 0.001

*Beta* coefficient, *SE* standard error

Table 5 shows the results of the Pearson correlation test, which shows the relationship between the sub-dimensions and total scores of the Yale food addiction and Motives for Caffeine Consumption Questionnaire. When the Pearson correlation test results were examined, it was found that the total scores of the individuals’ Motives for Caffeine Consumption Questionnaire and the Yale Food Addiction Scale sub-dimensions and total scores were respectively (*r* = 0.121, *p* > 0.05) as follows: no correlation, (*r* = 0.071, *p* > 0.05) no correlation, (*r* = 0.180, *p* < 0.05) low level in positive direction, (*r* = 0.138, *p* > 0.05) no correlation, (*r* = − 0.224, *p* < 0.05) low level in negative direction, (*r* = − 0.143, *p* < 0.05) low level in negative direction, (*r* = 0.280, *p* < 0.05) low level in positive direction, (*r* = 0.158, *p* < 0.05) low level in positive direction, and (*r* = 0.172, *p* < 0.05) low level in positive direction.

**Table 5:** The relationship between the sub-dimensions and total scores of the Yale food addiction and caffeine consumption motivation scales of individuals

	**Caffeine total**	**Habit**	**Alertness**	**Mood**	**Social**	**Taste**	**Symptom management**
**TSEE**	0.121	0.051	0.107	0.096	0.221^*^	0.081	0.081
**ORR**	0.071	0.115	0.087	− 0.017	− 0.084	0.116	0.120
**STMTP**	0.180^*^	0.099	0.197^*^	0.130	0.334^*^	0.036	0.161^*^
**GDS**	0.138	0.069	0.125	0.067	0.202^*^	0.033	0.249^*^
**CUBR**	− 0.224^*^	− 0.200^*^	− 0.189^*^	− 0.217^*^	− 0.270^*^	− 0.092	− 0.152^*^
TD	− 0.143^*^	− 0.078	− 0.148^*^	− 0.129	− 0.269^*^	− 0.027	− 0.102
CWS	0.280^*^	0.242^*^	0.280^*^	0.188^*^	0.309^*^	0.195^*^	0.208^*^
UCCI	0.158^*^	0.113	0.100	0.091	0.280^*^	0.100	0.141^*^
Yale total	0.172^*^	0.116	0.167^*^	0.072	0.218^*^	0.121	0.207^*^

*TSEE* taking the substance in excess for longer than expected, *ORR* ongoing request or repeated failures to quit, *STMTP* spending too much time in procurement, use, and overcoming, *GDS* giving up or decreasing social, occupational, and leisure activities, *CUBR* continuing to use even though it is known to have bad results, *TD* tolerance development (in effect decrease, increase in amount), *CWS* characteristic withdrawal symptoms, substance use to reduce withdrawal symptoms, *UCCI* use to cause clinically significant impairment

^*^p < 0.05

It was seen that there was no correlation (*r* = 0.051, *p* > 0.05), no correlation (*r* = 0.115, *p* > 0.05), no correlation (*r* = 0.099, *p* > 0.059), no correlation (*r* = 0.069, *p* > 0.05), low level in negative direction (*r* = − 0.200, *p* < 0.05), no correlation (*r* = − 0.078, *p* > 0.05), low level in positive direction (*r* = 0.242, *p* < 0.05), no correlation (*r* = 0.113, *p* > 0.05), and no correlation (*r* = 0.116, *p* > 0.05) between habit subscale scores and Yale food addiction scale sub-dimensions and total scores, respectively.

It was determined that there was no correlation (*r* = 0.107, *p* > 0.05), no correlation (*r* = 0.087, *p* > 0.05), low level in positive direction (*r* = 0.197, *p* < 0.05), no correlation (*r* = 0.125, *p* > 0.05), low level in negative direction (*r* = − 0.189, *p* < 0.05), low level in negative direction (*r* = − 0.148, *p* < 0.05), negative low level in positive direction (*r* = 0.280, *p* < 0.05), no correlation (*r* = 0.100, *p* > 0.05), and low level in positive direction (*r* = 0.167, *p* < 0.05) between the wakefulness sub-dimensions scores and the Yale food addiction scale sub-dimensions and total scores, respectively.

It was determined that there was no correlation (*r* = 0.096, *p* > 0.05), no correlation (*r* = − 0.017, *p* > 0.05), no correlation (*r* = 0.130, *p* > 0.05), no correlation (*r* = 0.067, *p* > 0.05), low level in the negative direction (*r* = − 0.217, *p* < 0.05), no correlation (*r* = − 0.129, *p* > 0.05), low level in the positive direction (*r* = 0.188, *p* < 0.05), no correlation (*r* = 0.091, *p* > 0.05), and no correlation (*r* = 0.072, *p* > 0.05) between mood sub-dimension scores and Yale food addiction scale sub-dimensions and total scores, respectively.

There is a relationship that low level in the positive direction (*r* = 0.221, *p* < 0.05), no correlation (*r* = − 0.084, *p* > 0.05), medium level in the positive direction (*r* = 0.334, *p* < 0.05), low level in the positive direction (*r* = 0.202, *p* < 0.05), low level in the negative direction (*r* = − 0.270, *p* < 0.05), low level in the negative direction (*r* = − 0.269, *p* < 0.05), medium level in the positive direction (*r* = 0.309, *p* < 0.05), low level in positive direction (*r* = 0.280, *p* < 0.05), and low level in positive direction (*r* = 0.218, *p* < 0.05) between social sub-dimension scores and Yale food addiction scale sub-dimensions and total scores, respectively,

It was determined that there was no correlation (*r* = 0.081, *p* > 0.05), no correlation (*r* = 0.116, *p* > 0.05), no correlation (*r* = 0.036, *p* > 0.05), no correlation (*r* = 0.033, *p* > 0.05), no correlation (*r* = − 0.092, *p* > 0.05), no correlation (*r* = − 0.027, *p* > 0.05), low level in the positive direction (*r* = 0.195, *p* < 0.05), no correlation (*r* = 0.100, *p* > 0.05) relationship, and no correlation (*r* = 0.121, *p* > 0.05) between taste sub-dimension scores and Yale food addiction scale sub-dimensions and total scores, respectively.

It was determined that there was no correlation (*r* = 0.081, *p* > 0.05), no correlation (*r* = 0.120, *p* > 0.05), low level in the positive direction (*r* = 0.161, *p* < 0.05), low level in the positive direction (*r* = 0.249, *p* < 0.05), low level in the negative direction (*r* = − 0.152, *p* < 0.05), no correlation (*r* = − 0.102, *p* > 0.05), low level in the positive direction (*r* = 0.208, *p* < 0.05), low level in the positive direction (*r* = 0.141, *p* < 0.05), and low level in the positive direction (*r* = 0.207, *p* < 0.05) between symptom management sub-dimension scores and Yale food addiction scale sub-dimensions and total scores, respectively.

## Discussion and conclusion

In this study, it was aimed to perform the Turkish validity and reliability study of the MCCQ, which was developed by Ágoston et al. (2018), as a tool to determine the habit, alertness, mood, influence of the social environment, taste, and symptom management on the caffeine consumption motivation in young adults. Confirmatory factor analysis, Pearson correlation test, and Cronbach’s alpha coefficient were used to perform the Turkish validity and reliability analysis of the scale. As a result of the analysis, the final scale consisting of 23 items and 6 sub-dimensions was found to be valid and reliable to measure the caffeine consumption motivation in the Turkish sample. As a result of the confirmatory factor analysis of the original English scale, it was reported that *CFI* = 0.912 and *RMSEA* = 0.074, among the fit indices, were at acceptable levels (Ágoston et al. 2018). In the scale we adapted into Turkish, *χ*^2^/*SD* = 0.752 and *CFI* = 1.000 were found to be excellent, and *GFI* = 0.991 and *RMSEA* = 0.000 were found to be acceptable. When the English original values of the scale are compared with the reference values we use, it is seen that the scale is at an excellent level. It is possible to say that the fit indices are similar to each other when compared with the scale we adapted into Turkish. It was reported as 0.81 for habit sub-dimension, 0.95 for alertness sub-dimension, 0.86 for mood sub-dimension, 0.91 for social sub-dimension, 0.91 for taste sub-dimension and 0.66 for symptom management sub-dimension of the English main scale (Ágoston et al. 2018). It was calculated as 0.96 for the habit sub-dimension, 0.86 for the alertness sub-dimension, 0.92 for the mood sub-dimension, 0.88 for the social sub-dimension, 0.91 for the taste sub-dimension, and 0.58 for the symptom management sub-dimension of the Turkish scale. For both scales, it is seen that the internal consistency for habit, alertness, mood, social, and taste sub-dimensions is highly reliable, while the internal consistency of the symptom management sub-dimension is low.

The studies-proven benefits of caffeine include increased alertness, endurance and attention, and combating fatigue. While these desirable effects are likely to potentiate caffeine consumption, adverse effects such as anxiety, irritability, and sleep disturbances may also appear to prevent additional or continued caffeine consumption (Stachyshyn et al. 2021). It has been stated in the literature that caffeine can affect human health in both positive and negative ways. It has been reported that caffeine can improve many cognitive and behavioral processes such as exercise level, fatigue, and concentration and reduce fatigue with the stimulant effect of moderate caffeine intake (Zahra et al. 2020). Caffeine has been classified under the “generally recognized as safe” (GRAS) category by the US Food and Drug Administration (FDA) (Rosenfeld et al. 2014). Caffeine intake of ≤ 200 mg/day in a single dose and ≤ 400 mg/day in total for a healthy adult was accepted as safe by European Food Safety Authority (EFSA), and it was stated that no adverse side effects would be expected (EFSA Scientific Panel NDA, 2015).

In conclusion, our study shows that the Turkish version of the Motives for Caffeine Consumption Questionnaire is reliable (Table 5). We believe that the Motives for Caffeine Consumption Questionnaire can be used to evaluate the reasons for caffeine consumption of individuals. The correlations found work as expected as they reflect the relationship between the data statistically accurately enough. This scale was developed as a tool to assess motivation for caffeine consumption and can make important contributions in various disciplines. Academic researchers can use this scale to delve deeper into studies of caffeine addiction and assess the risk of addiction. Likewise, it can guide researchers who want to understand the health effects of caffeine consumption in health and clinical research, identify individuals’ caffeine habits in nutrition and dietary studies, and investigate psychological models of addiction. This scale can also help health professionals to develop counseling or treatment strategies related to caffeine dependence or undesirable habits. In conclusion, this scale can be considered as a qualified tool that can make an important contribution to research or application studies in many different areas related to caffeine.

### Limitations of the study

Firstly, the study outnumbered women. Secondly, the findings of this study are based on self-report data, which carries the risk of source bias. In further research, multiple assessment methods can be used (e.g., experimental paradigms, clinical practice). Thirdly, the sample power analysis result evaluated in this study was sufficient, but data collection took place online. Data collected during the pandemic; therefore, data collection was conducted online to minimize the risk of infection. The sample may be limited in its ability to access all strata of the population (for example, individuals without Internet). Its suitability for use in clinical and research settings (via clinical practice and/or other modes of application such as face-to-face interviews) and in a wider variety of populations, including clinical specimens, should be further explored in future studies. In future studies, the MCCQ can be adapted to individuals from different groups in the adult Turkish population.

## Data Availability

The datasets used and/or analyzed during the current study are available from the corresponding author on reasonable request.

## Abbreviations

GRAS: Generally recognized as safe
FDA: US Food and Drug Administration
EFSA: European Food Safety Authority
MCCQ: Motives for Caffeine Consumption Questionnaire
YFAS: Yale Food Addiction Scale
